# Cocultures of human colorectal tumor spheroids with immune cells reveal the therapeutic potential of MICA/B and NKG2A targeting for cancer treatment

**DOI:** 10.1186/s40425-019-0553-9

**Published:** 2019-03-14

**Authors:** Tristan Courau, Julie Bonnereau, Justine Chicoteau, Hugo Bottois, Romain Remark, Laura Assante Miranda, Antoine Toubert, Mathieu Blery, Thomas Aparicio, Matthieu Allez, Lionel Le Bourhis

**Affiliations:** 1 INSERM U1160, Institut de Recherche Saint-Louis, Saint Louis Hospital, Paris, France; 2 Paris-Diderot University, Sorbonne Paris Cité, Paris, France; 3 Gastroenterology and Digestive Oncology Department, Saint Louis Hospital, AP-HP, Paris, France; 4 Innate Pharma, Marseille, France

**Keywords:** Immunotherapy, Colorectal cancer, Spheroids, NKG2A, MICA/B

## Abstract

**Background:**

Immunotherapies still fail to benefit colorectal cancer (CRC) patients. Relevant functional assays aimed at studying these failures and the efficacy of cancer immunotherapy in human are scarce. 3D tumor cultures, called tumor organoids or spheroids, represent interesting models to study cancer treatments and could help to challenge these issues.

**Methods:**

We analyzed heterotypic cocultures of human colon tumor-derived spheroids with immune cells to assess the infiltration, activation and function of T and NK cells toward human colorectal tumors in vitro.

**Results:**

We showed that allogeneic T and NK cells rapidly infiltrated cell line-derived spheroids, inducing immune-mediated tumor cell apoptosis and spheroid destruction. NKG2D, a key activator of cytotoxic responses, was engaged on infiltrating cells. We thus assessed the therapeutic potential of an antibody targeting the specific ligands of NKG2D, MICA and MICB, in this system. Anti-MICA/B enhanced immune-dependent destruction of tumor spheroid by driving an increased NK cells infiltration and activation. Interestingly, tumor cells reacted to immune infiltration by upregulating HLA-E, ligand of the inhibitory receptor NKG2A expressed by CD8 and NK cells. NKG2A was increased after anti-MICA/B treatment and, accordingly, combination of anti-MICA/B and anti-NKG2A was synergistic. These observations were ultimately confirmed in a clinical relevant model of coculture between CRC patients-derived spheroids and autologous tumor-infiltrating lymphocytes.

**Conclusions:**

Altogether, we show that tumor spheroids represent a relevant tool to study tumor-lymphocyte interactions on human tissues and revealed the antitumor potential of immunomodulatory antibodies targeting MICA/B and NKG2A.

**Electronic supplementary material:**

The online version of this article (10.1186/s40425-019-0553-9) contains supplementary material, which is available to authorized users.

## Background

T cells and Natural Killer (NK) cells are major effectors of antitumor immune responses [1, 2]. Immunotherapies aimed at enhancing their activity have now reached bedside, with some outstanding effects reported in melanoma, breast or kidney cancers [3, 4]. Nevertheless, large proportions of patients fail to respond to these treatments [5] while other tumor types such as colorectal (CRC) and pancreatic cancers remain poorly responsive to immunomodulation [6]. There is thus a need to study the mode of action of existing immunotherapeutic agents and to dissect the cellular and molecular events underlying failures. This will allow the development of new immunomodulators for non-responsive cancers.

One of the challenges resides in the development of relevant models to human pathologies. Indeed, mice models hardly mimic treatment resistance observed in patients and human models mainly consist of monolayer cultures that poorly recapitulate the complex features of tumor environment [7, 8]. Recently, three-dimensional tumor cultures, called tumor organoids or spheroids, have been developed from tumor cell lines or primary tumor samples cultured in non-adherent conditions [8–11]. Many studies have highlighted the similarities of these spheroids to human tumors regarding their necrotic and proliferative zones, physicochemical parameters or maintenance of mutated pathways [12–15]. Consequently, spheroids are now widely used to study the efficacy of cytotoxic treatments in various cancer types [16–19].

Interactions of tumor spheroids with T and NK cells started to be explored through heterotypic cocultures [20]. These studies brought information about NK [21–25] and T cells [26–32] capacity to infiltrate and kill tumor spheroids in various context. Yet, they did not deepen the characterization of the infiltrating cells, the mechanisms of tumor-lymphocytes interactions through activating or inhibitory pathways, nor the impact of immune modulators on tumor spheroids fate.

NK cell receptors provide activating or inhibitory signals to induce direct cytotoxicity, antibody-dependent cellular cytotoxicity (ADCC) or tolerance directed against target cells. Among them, NKG2D and NKG2A are expressed by NK cells, significant proportions of CD8 T cells and subsets of CD4 T cells [33–36]. Interactions between NKG2D and its ligands MICA/B activate NK cell cytotoxicity and have been reported to be an important costimulatory signal for T cells [37, 38]. NKG2A binding to the MHC-like molecule HLA-E provides powerful inhibitory signaling in both T and NK cells [39, 40]. NKG2D-MICA/B and NKG2A-HLA-E pathways have been reported by our group and others as engaged in CRC and could be a potential immunotherapy for patients with CRC [41–45].

We therefore developed cocultures of CRC tumors and immune cells to study the infiltration, activation and function of immune cells toward human tumors with a particular focus on NKG2D and NKG2A pathways. We set up this model in allogeneic conditions, using healthy donor blood cells (HD PBMCs) and HT29 tumor cell line, and more importantly in autologous conditions using primary tumor-derived spheroids and tumor infiltrating lymphocytes (TILs) from the same patients.

We showed that activated/memory T and NK cells were able to infiltrate spheroids, kill tumor cells and disrupt the three-dimensional structure. Both NKG2D-MICA/B and NKG2A-HLA-E pathways were involved in these processes. Accordingly, we showed that anti-MICA/B antibodies efficiently stimulated antitumor responses. While NKG2A blockade did not show significative impact alone, combination of both MICA/B and NKG2A targeting was synergistic, inducing a strong immune-dependent destruction of patients-derived spheroids during cocultures with autologous TILs.

## Methods

### Cell line

Mycoplasma-free HT-29 and DLD1 tumor cell lines were obtained from ATCC (cat. HTB-38 and CCL-221) and cultured in HEPES-containing RPMI 1640 (ThermoFisher) complemented with 10% FCS (Eurobio), 50 U/mL penicillin, 50 μg/mL streptomycin, 2 mM GlutaMAX and 1 mM Sodium Pyruvate (all from ThermoFisher), thereafter named complete RPMI.

### Healthy donors (HD) blood cells

Heparinized venous blood was collected from healthy donors, diluted 1:2 with PBS and then layered on a density gradient (Lymphocytes separation medium, Eurobio). Peripheral blood mononuclear cells (PBMCs) were collected from the interface after centrifugation, washed with PBS, and resuspended in complete RPMI.

### Cell sorting

T and NK cells were enriched from HD PBMCs by magnetic cell depletion of B cells and monocytes using anti-CD19 and anti-CD14 microbeads with the MACS technology (Miltenyi), according to the manufacturer procedures. Particular CD4, CD8, and NK cells enrichments were respectively achieved by positively sorting CD4+, CD8+ and CD8-CD56+ cells from HD PBMCs with the MACS technology.

### Coculture protocol

HT29 (or DLD1) spheroids were generated by seeding 10^4^ cells per well on Nunclon Sphera (ThermoFisher) or Costar ultra-low attachment (Corning) round bottom 96 wells plates in complete RPMI. 5 days later, spheroids contained 3. 10^4^ cells and cocultures were started by adding 3.10^5^ total or CD19^−^CD14^−^ sorted HD PBMCs per well, together with stimulatory or inhibitory molecules. In Fig. 3, 3.10^4^ cells of each subset were added with the spheroids to perform cocultures. For flow cytometry analyses, 6 wells per condition were seeded. OUT and IN compartments were isolated by first pooling the 6 cocultures wells in eppendorf tubes. Spheroids were gently resuspended and left to sediment to the bottom of the eppendorf. Supernatant cell suspension constituted the non-infiltrating immune cells (=OUT). These steps were repeated 2 times with PBS in order to wash the spheroids from the non-infiltrating immune cells. Spheroids were then trypsinized to obtain a single cell suspension (=IN) further analyzed by flow cytometry.

### Cytokines and functional antibodies

The following commercial stimulatory or inhibitory molecules were used in our cocultures: Interleukin 15 (IL-15, used at 10 ng/mL, Miltenyi), anti-IFNγ blocking antibodies (B27 clone, used at 2 μg/mL, BD Biosciences) and anti-NKG2D blocking antibodies (1D11 clone, used at 5 μg/mL, BD Biosciences). Anti-NKG2A (monalizumab, hIgG4) and ADCC-enhanced anti-MICA/B (IPH4301, hIgG1) were provided by Innate Pharma along with corresponding isotype controls.

### Antibodies and flow cytometry analyses

Cells were stained with saturating amounts of various fluorescent-labeled antibody combinations including anti-EpCAM (EBA-1 clone), anti-CD45 (HI30 clone), anti-CD3e (UCHT1 clone), anti-CD56 (B159 clone), anti-CD4 (SK3 clone), anti-CD8 (SK1 clone), anti-CD25 (M-A215 clone), anti-CD107a (H4A3 clone), anti-CD45RO (REA611 clone), anti-NKG2A (REA110 clone), anti-NKG2D (BAT221 clone), anti-CD137 (4B4–1 clone), anti-CD16 (3G8 clone), anti-HLA-E (3D12 clone), Annexin V and co-stained with DAPI (all from BD Biosciences or Miltenyi). Cells were analyzed with the Attune NxT flow cytometer (Life Technologies) and further analyses were performed with FlowJo software (Tree Star).

### Spheroid volume calculation

Before trypsinization, pooled spheroids were placed in 96 wells plate and pictured using the EVOS FLc microscope at a 2x to 4x magnification. Images were then analyzed using the Icy software by measuring the length (L) and width (W) of each spheroid. Spheroid volumes were then calculated as follows: V = (L x W x W) / 2.

### Live imaging of spheroid apoptosis

This was achieved by adding CellEvent caspase-3/− 7 green dye (ThermoFisher) in the coculture wells and imaging green fluorescence across time using the Incucyte S3 system (Essen BioScience). Single pictures of each well were acquired every hour during 1 week and analyzed using Incucyte S3 software.

### Immunofluorescence (IF)

Before starting the cocultures, CD19^−^CD14^−^ sorted HD PBMCs were stained with CFSE (ThermoFisher) according to manufacturer procedure. Infiltrated spheroids were isolated at 24 h and embedded in Tissue-Tek OCT compound (Sakura). Spheroid blocs were then 20 μm-thick sliced using CM1520 cryostat (Leica), the slides were mounted with DAPI-containing Fluoromount-G (Thermofisher) and imaged at a 5x magnification using an epifluorescence microscope (Axio Imager 2, Zeiss). Quantification of CFSE+ cells infiltrating the spheroids was done using the H-K means plugin of the Icy software.

### Immunohistochemistry (IHC)

The formalin-fixed spheroids were first embedded in Histogel (Thermo Scientific) and then in paraffin. Blocks were sliced in 5 μm-thick sections and immunostainings performed on a Discovery Ultra automaton (Ventana). After pre-treatment with cell conditioning 1 (Ventana), sections were incubated 1 h at 37 °C with anti-MICA/B (clone MIA4, Innate Pharma) or anti-HLA-E (clone MEM-E/02, Exbio) primary antibodies at 2 μg/mL and 1 μg/mL, respectively. Anti-mouse IgG detection system (discovery OmniMap anti-mouse HRP, Ventana) was used for HLA-E staining and an additional amplification step using tyramide was used for MICA/B staining (discovery Amp HQ kit, Ventana). After revelation with 3,3-diaminobenzidine and counterstaining with hematoxylin, sections were washed, dehydrated, cleared and mounted using a coverslipper (ClearVue, Thermo Scientific). Stained sections were finally scanned on a slide scanner (S60 Nanozoomer, Hamamatsu).

### Colorectal cancer patients (CRC) samples

Between February 2017 and February 2018, 41 patients who underwent resection of colon cancer at the Saint Louis Hospital in Paris were prospectively included. This study was approved by the French ethical committee (approval n°2016/45), and all subjects gave written informed consent.

Patients’ blood was manipulated the same way than HD blood (see above). Tumor samples were washed with PBS (ThermoFisher) and cut into pieces of 2 to 3 mm. Fragments were incubated for 20 min at room temperature with an antibiotic cocktail containing fungizone, normocin and gentamicin (all from ThermoFisher) to avoid contamination and then enzymatically digested for a total of 21 min at 37 °C in complete RPMI containing collagenase IV (Sigma-Aldrich) and DNase I (Roche). The supernatant was then filtered and the immune cells extracted with lymphocyte separation medium (Eurobio). Resulting tumor-infiltrating lymphocytes (TILs) were either directly analyzed by flow cytometry or cultured in complete RPMI complemented with IL-15 and IL-7 (10 ng/mL, both from Mitenyi) in order to keep them alive before coculture starting with autologous tumor spheroids.

Generation of patient-derived tumor spheroids was achieved by first culturing digested and filtered tumor in classic adherent culture flasks (BD Falcon) in complete RPMI. Cells were then washed every 2 days and cultured for up to 10 days. This allowed the isolation of adherent cells that were then trypsinized and seeded in ultra-low attachment 96 wells plates to create a unique uniform spheroid in each well. Cell numbers and subsequent setup and analyses of autologous cocultures were done using the same protocol than cocultures between HT29 cell line and HD PBMCs.

### Statistical analyses

Data are expressed as Mean ± SEM. Statistically significance of differences was analyzed using GraphPad Prism 7 (GraphPad Software, La Jolla, USA) by paired Student’s *t* test, two-way ANOVA or Wilcoxon matched-pairs signed rank test when appropriate. A *p* value < 0.05 was considered as statistically significant.

## Results

### Activated/memory T cells and NK cells infiltrate colon cancer cell line-derived spheroids

**Fig. 1:**
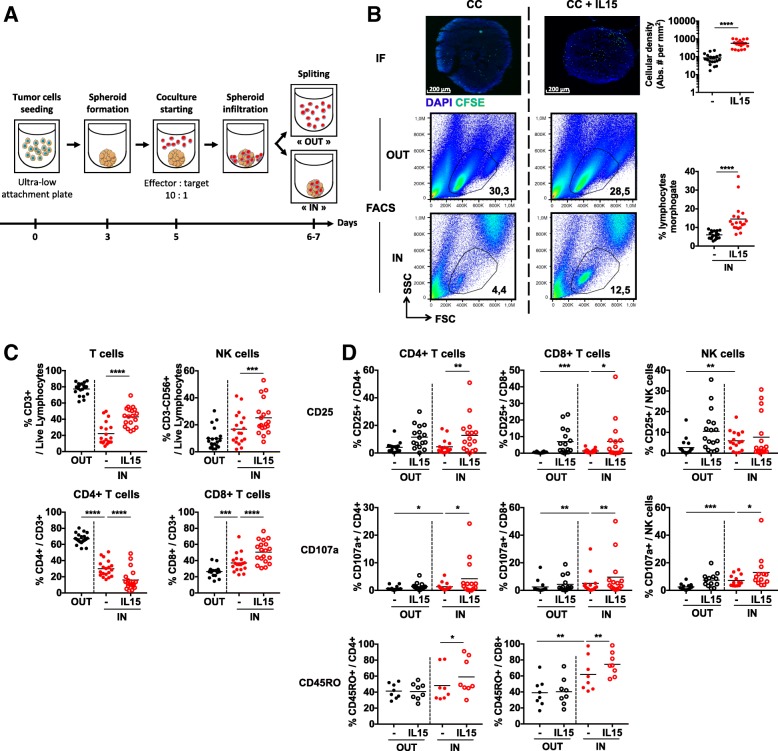
Allogeneic activated/memory T and NK cells are able to infiltrate HT29 tumor spheroids. **a** Scheme of the coculture (CC) protocol between HT29 spheroids and CD19-CD14- sorted PBMCs from healthy donors. **b** Immunofluorescence (*n* = 2 independent experiments) and flow cytometry (*n* = 19 independent experiments) analyses of spheroid immune infiltration in the presence or not of IL-15 at 24 h. **c** Flow cytometry analyses of T and NK cells (respectively gated CD3+ and CD3-CD56+ among live single cells lymphocytes) as well as CD4+ and CD8+ T cells subsets (respectively gated CD4 + CD8- and CD4-CD8+ among CD3+) percentages in the IN and OUT compartments, in the presence or not of IL-15 at 24 h. *n* = 19 independent experiments. **d** Flow cytometry analyses of CD25, CD107a and CD45RO expression by CD4+ T cells, CD8+ T cells and NK cells in the IN and OUT compartments in the presence or not of IL-15 at 24 h. *n* = 8 to 18 independent experiments. Statistical significance of immunofluorescence experiments was analyzed using the Mann-Whitney test, the others using Wilcoxon matched-pairs signed rank test (* *p* < 0.05; ** *p* < 0.005, *** *p* < 0.001, **** *p* < 0.0001)

We generated colon cancer spheroids from HT29 cell line that we cocultured with peripheral blood immune cells from healthy donors (HD PBMCs), depleted of B cells and monocytes in order to enrich for T and NK cells. After coculture, infiltrating cells (IN) and cells remaining in the medium (OUT) were mechanically separated and analyzed (Fig. 1a).

To assess whether immune cells entered in the spheroids or remained in the culture medium, we analyzed cocultured spheroids by immunofluorescence (IF) and observed a deep and homogeneous infiltration of pre-stained immune cells (Fig. 1b, upper panels). By flow cytometry, we could detect infiltrating cells 24 h after coculture initiation (Fig. 1b, bottom panels), reaching about 1500 total immune cells per spheroid. Comparing cellular proportions IN and OUT of the spheroids, we observed lower T cell and higher NK cell proportions in the tumor structure (Fig. 1c, upper panels). We also found a reversal of CD4 to CD8 subpopulations ratio in the spheroids (Fig. 1c, bottom panels), pointing that NK and CD8 T cells could have a particular advantage for spheroid infiltration.

Infiltrating CD8 T cells showed increased proportions of CD45RO+ memory cells. Additionally, NK and CD8 T cells displayed increased expression of the activation marker CD25 and of the degranulation marker CD107a in the spheroids compared to OUT cells (Fig. 1d). Conversely, CD4 T cells phenotype did not seem to be impacted by spheroid infiltration, with no change in memory or activation markers observed inside the spheroids. CD4 Tregs proportions were found unchanged as well in this context (Additional file 1: Figure S1). These results suggest that memory CD8 T cells and NK cells are prone to infiltrate and get activated in spheroids.

### Immune stimulation increases HT29 spheroid infiltration

We tested the possibility to modulate the immune response in this system by adding IL-15 to the cocultures. IL-15 induced a strong increase of spheroid infiltration by both T and NK cells (Fig. 1b), reaching up to 4500 total immune cells infiltrated per spheroid. It also increased the proportions of infiltrating CD8 T cells (Fig. 1c) and enhanced the activation of all infiltrating cells, as witnessed by increased proportions of CD45RO+ memory CD4 and CD8 T cells and increased expression of CD25 and CD107a by T and NK cells in the spheroids (Fig. 1d). This shows that known immune mediators, such as IL-15, can modulate the activity of T and NK cells within HT29 spheroids.

### Spheroid infiltration leads to tumor cell apoptosis and spheroid destruction

**Fig. 2:**
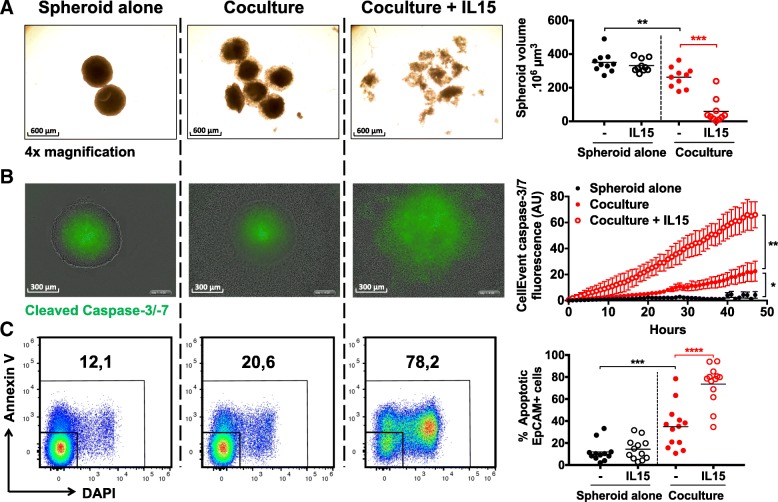
Immune infiltration and activation in HT29 spheroids lead to tumor cell apoptosis and spheroid destruction. HT29 spheroids were cocultured or not with CD19-CD14- PBMCs and IL-15 and we monitored **a** spheroid volume using microscopic pictures, **b** the dynamic cleavage of caspase-3/− 7 in the spheroids using Incucyte live imaging system, and **c** the staining by Annexin V and DAPI dyes of EpCAM+ cells by flow cytometry at 48 h. *n* = 4 to 12 independent experiments. Statistical significance of A panel was analyzed using 2-way ANOVA, the other panels using the Wilcoxon matched-pairs signed rank test (* *p* < 0.05; ** *p* < 0.005, *** *p* < 0.001, **** *p* < 0.0001)


We explored the functional consequences of this immune cell infiltration and activation in spheroids using several methods. We first examined spheroid integrity by monitoring their volume through picture-based measurements (Fig. 2a). HT29 spheroids cocultured with T and NK cells-enriched HD PBMCs were smaller than control spheroid at 48 h, suggesting that immune cells disrupted the tumor structure (Fig. 2a). To examine the cause of such disruption, we assessed the global activation of caspase-3 and -7 in the spheroids by live-imaging (Fig. 2b and Additional file 2: Movie S1, Additional file 3: Movie S2, Additional file 4: Movie S3) and quantified Annexin V and DAPI staining of tumor cells by flow cytometry (Fig. 2c). These complementary analyses showed that spheroid destruction was correlated to an active apoptosis process in tumor cells, involving caspase-3 and -7 activation, which resulted in the accumulation of AnnexinV+DAPI+ apoptotic tumor cells.

**Additional file 2: Movie S1.** Spheroid alone. (MP4 27372 kb)



**Additional file 3: Movie S2.** Spheroid and Immune cell Coculture. (MP4 36506 kb)



**Additional file 4: Movie S3.** Spheroid and Immune cell Coculture with IL-15. (MP4 36436 kb)


IL-15 enhanced these apoptotic processes and induced an almost complete spheroid destruction at 48 h (Fig. 2a, b and c). Conversely, IFNγ blockade in our system resulted in decreased spheroid destruction, tumor cell apoptosis and spheroid infiltration by immune cells compared to control conditions (Additional file 1: Figure S2). Similar results were obtained using another colon cell line, DLD-1 (data not shown). Together, these results show that infiltration of tumor spheroid by activated/memory immune cells triggers tumor cell killing and spheroid destruction, which can be enhanced or dampened by immune modulation.

### CD4 T cells, CD8 T cells and NK cells are self-sufficient to exert antitumor effects during the cocultures

**Fig. 3:**
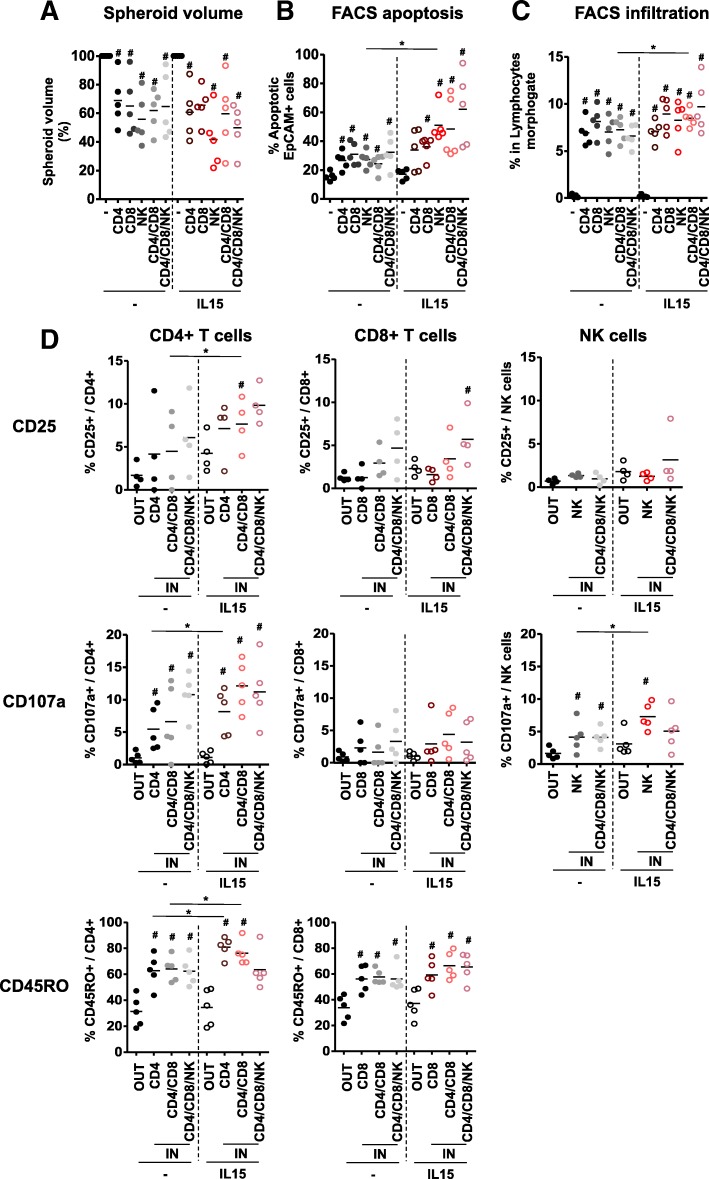
Specific effects of CD4, CD8 and NK cells cocultured with HT29 spheroids alone or combined. HT29 spheroids were cocultured with sorted CD4 T cells, CD8 T cells and NK cells at a 1:1 ratio, alone or in combination and with or without IL-15. We analyzed **a** spheroid destruction through analysis of macroscopic pictures and **b** tumor cell apoptosis using flow cytometry after 48 h as well as **c** immune infiltration and **d** expression of CD25, CD107a and CD45RO by CD4 T cells, CD8 T cells and NK cells in IN and OUT compartments after 24 h. *n* = 4 to 5 independent experiments. Statistical significance was analyzed using the Wilcoxon matched-pairs signed rank test (# *p* = 0.06, as compared to spheroid alone in A to C panels and to OUT cells in D panels)

To test the intrinsic capacity of CD4 T cells, CD8 T cells and NK cells to react toward tumor spheroids we purified and cocultured them, alone or combined, with HT29 tumor spheroids. We observed that the three cell types were self-sufficient to disrupt the three-dimensional structure, induce tumor cell apoptosis and infiltrate tumor spheroids (Fig. 3a to c). In this context, infiltrating cells showed increased levels of CD25 and CD107a compared to cells outside the spheroid, as well as higher proportion of CD45RO expression in T cells (Fig. 3d). This demonstrates the capacity of the three cell types to react to the tumor cells upon infiltration, and confirms our previous observations of an increased infiltration capacity of memory T cells as compared to naïve T cells.

Overall, we also observed that NK cells were prompter than T cell subsets to infiltrate spheroids and kill tumor cells, but also that combination of the three cell types had an additive impact on the particular activation of each of them. This implies a possible cellular cooperation process between T cells and NK cells to destroy tumor spheroids in our model.

Adding of IL-15 during these cocultures then allowed us to refine our previous conclusions by showing that IL-15-mediated effects were mainly driven by NK cells in this context. However, IL-15 increased tumor cell apoptosis induced by the combination of CD4 and CD8 T cells, implying that IL-15 is not exclusively sensed and active through NK cells.

### NKG2D-MICA/B pathway is engaged during the cocultures

**Fig. 4:**
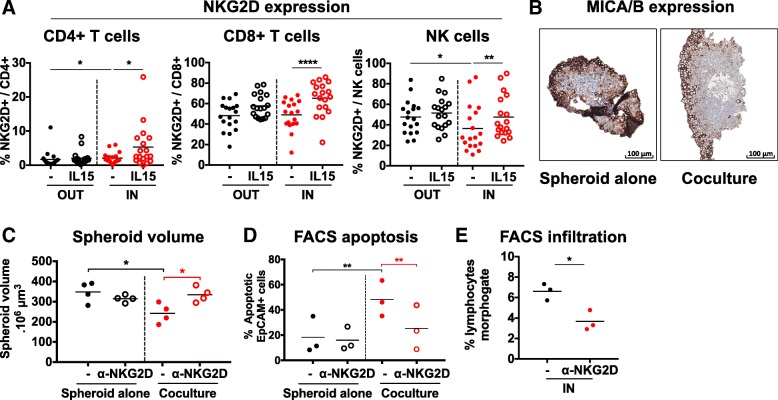
NKG2D-MICA/B pathway is engaged during the cocultures. **a** NKG2D expression by CD4 T cells, CD8 T cells and NK cells in the IN and OUT compartments, in the presence or not of IL-15, as measured by flow cytometry at 24 h. *n* = 18 independent experiments **b** MICA/B expression by tumor cells in the spheroids cocultured or not with CD19-CD14- PBMCs, as measured by immunohistochemistry at 24 h. Representative pictures of 1 experiment. **c** to **e** Analyses of **c** spheroid volume, **d** tumor cell apoptosis and **e** spheroid infiltration 48 h after coculturing HT29 spheroids with CD19-CD14- PBMCs in the presence or not of anti-NKG2D blocking antibodies. *n* = 3 to 4 independent experiments. Statistical significance of A panel was analyzed using the Wilcoxon matched-pairs signed rank test, C to E panels using paired t test (* *p* < 0.05; ** *p* < 0.005, *** *p* < 0.001, **** *p* < 0.0001)

We observed that NKG2D expression was increased on infiltrating CD4 T cells, unchanged on CD8 T cells and decreased on NK cells (Fig. 4a), advocating for an engagement of NKG2D in the spheroids [43]. In parallel, immunohistochemistry (IHC) analyses showed strong expression of NKG2D ligand MICA/B on tumor cells in both control and cocultured spheroids (Fig. 4b).

We tested the functional relevance of NKG2D-MICA/B pathway by using anti-NKG2D blocking antibodies in the cocultures. Spheroid infiltration by immune cells as well as spheroid destruction and tumor cell apoptosis were significantly decreased in this condition (Fig. 4c, d and e, respectively), suggesting that MICA/B are major targets of cytotoxic cells infiltrating spheroids.

### Anti-MICA/B antibodies exert antitumor effects

**Fig. 5:**
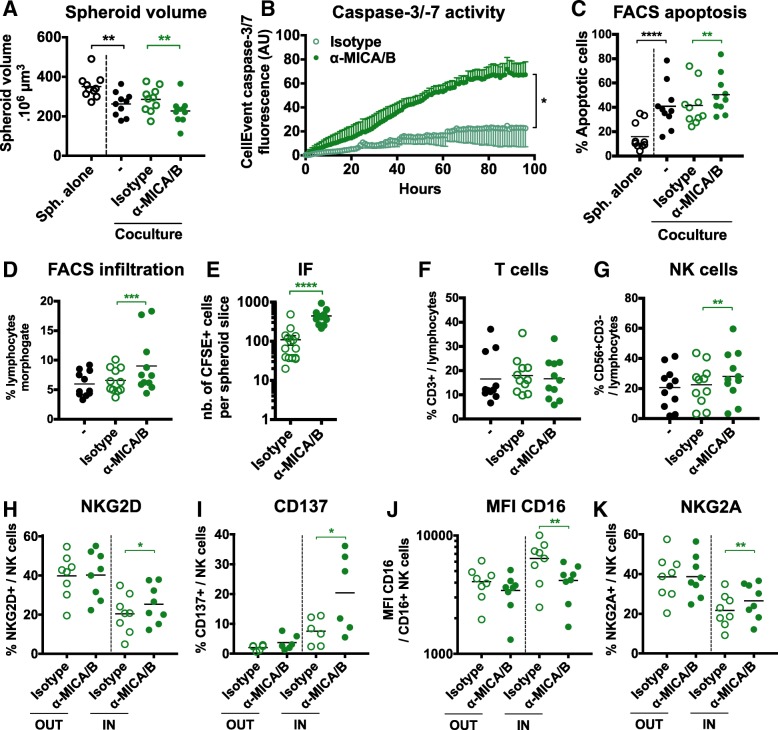
Anti-MICA/B induce immune-mediated anti-tumor effects through increased NK cell infiltration and activation in HT29 spheroids. HT29 spheroids were cocultured or not with CD19-CD14- PBMCs in the presence or not of anti-MICA/B antibodies or corresponding control isotype. We analyzed tumor cell death and spheroid destruction using (**a**, *n* = 10) microscopic pictures at 48 h, (**b**, *n* = 4) Incucyte live imaging or (**c**, *n* = 10) flow cytometry at 48 h, as well as (**d** and **e**) overall or (**f** and **g**) particular T and NK cells infiltration using flow cytometry (**d**, **f** and **g**, *n* = 10) and immunofluorescence (**e**, *n* = 1) at 24 h. We also analyzed by flow cytometry the expression of (**h**, *n* = 8) NKG2D, (**i**, *n* = 6) CD137, (**j**, *n* = 7) CD16 and (**k**, *n* = 8) NKG2A by NK cells in the IN and OUT compartments at 24 h. Statistical significance of immunofluorescence experiment was analyzed using the Mann-Whitney test, A panel using 2-way ANOVA and the other panels using the Wilcoxon matched-pairs signed rank test (* *p* < 0.05; ** *p* < 0.005, *** *p* < 0.001, **** *p* < 0.0001)

MICA/B expression by tumor cells and NKG2D pathway engagement prompted us to test the antitumor potential of an ADCC-enhanced anti-MICA/B antibody (Fig. 5). Compared to its control isotype, anti-MICA/B induced an increased immune-dependent spheroid destruction and tumor cell death, as observed by microscopic data, live-imaging and flow cytometry (Fig. 5a, b and c, respectively). No changes were observed in the absence of immune cells (not shown).

This tumor destruction was associated with an increase of overall spheroid infiltration by T and NK cells (Fig. 5d and e), with unchanged proportions of infiltrating T cells (Fig. 5f and Additional file 1: Figure S3A and B) but increased proportions of infiltrating NK cells (Fig. 5g). Interestingly, NKG2D expression on infiltrating NK cells was partially restored (Fig. 5h), confirming that anti-MICA/B also blocked NKG2D interaction with its ligands in this system (Innate Pharma unpublished results). Concurringly, CD8 T cells upregulated NKG2D inside the spheroids during anti-MICA/B treatment (Additional file 1: Figure S3C), indicating a possible engagement of NKG2D on CD8 T cells as well.

Anti-MICA/B induced a strong upregulation of the early activation marker CD137 on NK cells (Fig. 5i), advocating for their increased stimulation. The Fc receptor CD16 was strongly downregulated at the NK cell surface (Fig. 5j), exhibiting the engagement of this receptor during ADCC [46]. Taken together, these results show that anti-MICA/B antibodies induce a strong NK-mediated immune antitumor response in this spheroid model.

### NKG2A blockade enhances the antitumor effects of anti-MICA/B antibodies

**Fig. 6:**
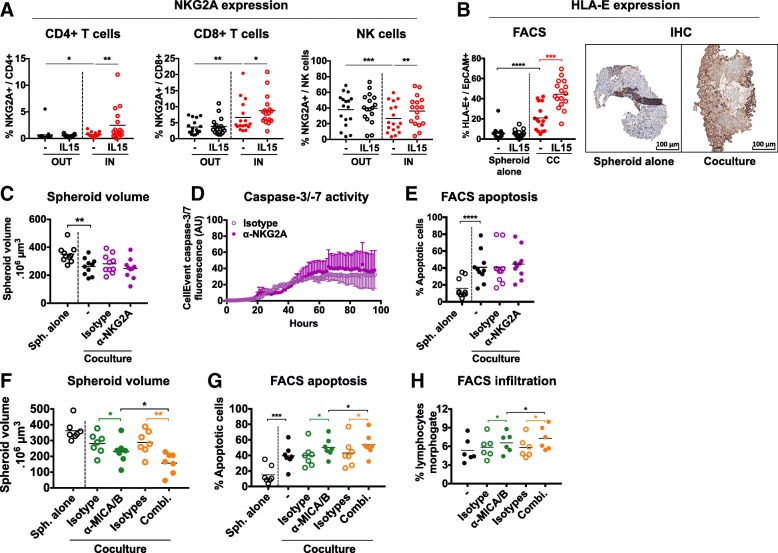
NKG2A-HLA-E pathway is engaged during the cocultures and NKG2A blockade synergizes with anti-MICA/B antibodies. **a** NKG2A expression by CD4 T cells, CD8 T cells and NK cells in the IN and OUT compartments and in the presence or not of IL-15, analyzed by flow cytometry at 24 h. *n* = 17 independent experiments. **b** HLA-E expression by tumor cells in the spheroids cocultured or not with CD19-CD14- PBMCs, analyzed by flow cytometry (*n* = 16 independent experiments) and immunohistochemistry (representative pictures of 1 experiment) at 24 h. **c** to **e** Analyses of (**c**, *n* = 4) caspase-3/− 7 activity, (**d**, *n* = 10) spheroid volume, and (**e**, *n* = 10) spheroid infiltration 48 h after coculturing HT29 spheroids with CD19-CD14- PBMCs in the presence or not of anti-NKG2A blocking antibodies or corresponding control isotype. **f** to **h** Analyses of (**f**, *n* = 7) spheroid volume, (**g**, *n* = 7) tumor cell apoptosis, and (**h**, *n* = 7) spheroid infiltration 48 h after coculturing HT29 spheroids with CD19-CD14- PBMCs in the presence or not of anti-MICA/B antibodies alone or combined with anti-NKG2A blocking antibodies, or with corresponding control isotype antibodies alone or combined. Statistical significance of **c** panel was analyzed using 2-way ANOVA, the other panels using the Wilcoxon matched-pairs signed rank test (* *p* < 0.05; ** *p* < 0.005, *** *p* < 0.001, **** *p* < 0.0001)

Interestingly, we observed an increased expression of the inhibitory receptor NKG2A on infiltrating NK cells after anti-MICA/B treatment (Fig. 5k). This led us to study the expression patterns of NKG2A and its ligand HLA-E during the cocultures. We first observed that NKG2A expression was increased on infiltrating T cells and downmodulated in infiltrating NK cells compared to OUT cells (Fig. 6a). Tumor cells did not express HLA-E in control spheroids but its expression was strongly induced by the presence of immune cells (Fig. 6b). Moreover, both NKG2A and HLA-E were significantly enhanced when immune cells were stimulated with IL-15 (Fig. 6a and b). This suggests that tumor cells in the spheroids could evade the immune response through NKG2A-HLA-E interactions.

We thus assessed the antitumor potential of an anti-NKG2A blocking antibody. Surprisingly, this antibody used alone did not exert antitumor effect in these conditions when looking at microscopic, live-imaging and flow cytometry readouts (Fig. 6c, d and e, respectively).

However, NKG2D and NKG2A pathways showed interesting interactions in our system, prompting us to test the efficacy of anti-MICA/B and anti-NKG2A combination. We observed increased spheroid alteration (Fig. 6f), tumor cells apoptosis (Fig. 6g) and spheroid infiltration (Fig. 6h) when anti-NKG2A was added to anti-MICA/B, compared to anti-MICA/B alone or isotype controls. No changes were observed in the absence of immune cells (not shown). These results suggest that anti-MICA/B and anti-NKG2A antibodies have a synergistic effect on immune mediated anti-tumor response in this spheroid model.

This effect was not linked to changes in T and NK cells proportions (Additional file 1: Figure S4A and B) but associated with an increased expression of CD137 by infiltrating CD8 T cells (Additional file 1: Figure S4C). These results confirm that NKG2A-HLA-E crosstalk is an interesting resistance mechanism to target in CRC and suggest that NKG2A additional blockade could reinforce CD8 T cells activation.

### NKG2A-HLA-E and NKG2D-MICA/B pathways are relevant targets in CRC patients

**Fig. 7:**
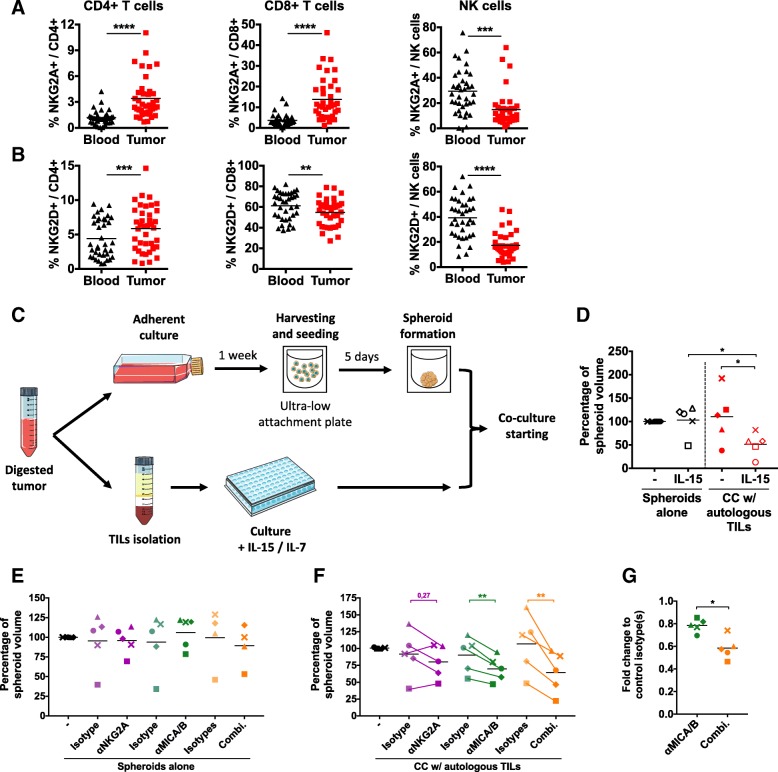
Cocultures between patients-derived spheroids and autologous TILs confirm therapeutic potential of MICA/B and NKG2A targeting. **a** and **b** Flow cytometry analyses of **a** NKG2A and **b** NKG2D expression by CD4 T cells, CD8 T cells and NK cells in the blood and tumor of colorectal cancer patients. *n* = 41 independent experiments. **c** Scheme of the protocol used to create primary colon tumor-derived spheroids and maintain autologous TILs in culture before cocultures. **d** Representation of the change in spheroid volume 48 h after coculturing or not primary CRC-derived spheroids and autologous TILs in the presence or not of IL-15, measured using microscopic pictures. **e** to **g** Change in spheroid volume 48 h after culturing primary CRC-derived spheroids with (**f** and **g**) or without (**e**) autologous TILs, in the presence or not of anti-MICA/B, anti-NKG2A or corresponding control isotypes. In **d** to **f** panels, spheroid volumes are normalized to the culture condition without stimulation. **g** panel represents fold changes between anti-MICA/B and its isotype, and between combination and combination of isotypes. In **d** to **g** panels, each patient is represented by a specific symbol. *n* = 5 independent experiments. Statistical significance of **a** and **b** panels was analyzed Wilcoxon matched-pairs signed rank test, the other panels using paired t test (* *p* < 0.05; ** *p* < 0.005, *** *p* < 0.001, **** *p* < 0.0001)

Our in vitro model suggests that the NKG2D-MICA/B pathway is a potential target in CRC treatment. Additionally, NKG2A-HLA-E interaction is a functional inhibitory mechanism arising during antitumor immune responses that could be targeted to improve treatment efficacy. To assess the relevance of these pathways in CRC, we investigated the expression of NKG2A and NKG2D on T and NK cells in the blood and the tumor of CRC-bearing patients (Fig. 7a and b).

We observed that T and NK cell populations presented respectively higher and lower levels of NKG2A in the tumor compared to blood (Fig. 7a). These results were comparable to what we observed between the OUT and IN compartments in the coculture model (Fig. 5a) and suggested that NKG2A pathway could be engaged in tumor-infiltrating cells during CRC.

NKG2D expression was also found lower on tumor-infiltrating CD8 T cells in CRC patients compared to blood, as already described [43]. This was also observed on NK cells (Fig. 7b), while NKG2D levels were found higher on tumor-infiltrating than on blood CD4 T cells. Intriguingly, these variations of NKG2D expression in patients were similar to those observed in our coculture model (Fig. 3a). Together, these results pointed to a potential functional relevance of NKG2A and NKG2D receptors in CRC patients.

### Tumor-infiltrating lymphocytes (TILs) do not exert antitumor effects against autologous CRC patient-derived spheroids without stimulation

To test this hypothesis, we derived primary CRC tumor-spheroids that we cocultured with autologous TILs (Fig. 7c and Additional file 1: Figures S5 and S6). In these settings we observed the impact of adding autologous TILs and/or IL-15 on spheroid volume. Interestingly, we showed that adding autologous TILs did not significantly impact the spheroid structure (Fig. 7d). However, IL-15 together with autologous TILs induced an immune-mediated destruction of tumor spheroids compared to control condition or IL-15 alone, showing that TILs stimulation can exert antitumor effect in these autologous conditions. We observed no correlation between the magnitude of these effects and respective clinical data nor tumor cell content in the spheroids, as well as TILs composition in T cells and NK cells (Additional file 1: Tables S1 and S2).

### Anti-MICA/B exert antitumor effect in autologous cocultures and synergizes with anti-NKG2A to disrupt tumor structure

We then tested the potential of anti-NKG2A and anti-MICA/B antibodies, alone or in combination, in these autologous conditions. Addition of these antibodies did not affect spheroid size in the absence of TILs (Fig. 7e), except for one patient for which spheroid growth was highly variable when cultured alone. During the cocultures anti-NKG2A alone did not show a reproducible effect while anti-MICA/B, alone or combined with anti-NKG2A, induced a significant immune-mediated decrease of spheroid size (Fig. 7f) compared to isotype control. By calculating the changes in spheroid volume induced by anti-MICA/B alone or combined with anti-NKG2A compared to their respective isotype control conditions, we also showed that anti-NKG2A increased the spheroid destruction induced by autologous TILs in the presence of anti-MICA/B antibodies.

These results show that anti-MICA/B antibodies are able to elicit an immune-mediated destruction of CRC patients-derived spheroids in an autologous context, and that additional targeting of NKG2A inhibitory receptor enhances this effect.

## Discussion

In this work, we describe that coculture of tumor spheroid with immune cells is a relevant and powerful tool to study human antitumor immune responses in vitro and ex vivo. We aimed at generating individual uniform spheroids in matrix- and growth factors-free medium to allow a rapid, precise and reproducible manipulation of coculture settings, including effectors to target cells ratio and treatments conditions. These models allowed us to develop innovative in vitro readouts such as measurements of tumor volume, shape and infiltration by immune cells as well as easy characterization of infiltrated cells, which cannot be achieved through classical monolayer culture models. By doing so, we showed that tumor spheroids can be infiltrated by allogeneic activated/memory T and NK cells which induce tumor cell death and spheroid destruction, notably through IFNγ and NKG2D-MICA/B pathways.

The latter has been identified for a long time as an important pathway in antitumor immunity but remained controversial in regard to its pro- or anti-tumorigenic role [47]. This controversy has been recently clarified by studies that identified NKG2D-MICA/B engagement as a major mechanism of tumor immune surveillance, subverted by tumors through proteolytic shedding of surface MICA/B which at the same time lower membrane-bound MICA/B recognition by NKG2D and induce the saturation of NKG2D with soluble MICA/B [37, 47]. Recent efforts to disrupt this tolerance mechanisms showed that inhibiting MICA/B shedding was a successful strategy to boost antitumor immune responses [48]. Our results support these concepts, by showing that NKG2D blockade prevents immune infiltration and activation in tumor spheroids and that exploiting MICA/B as a tumor antigen to induce ADCC is a feasible and efficient approach.

Interestingly, our results point at another resistance mechanisms used by tumor cells that try to evade immune recognition. This is illustrated by HLA-E upregulation on tumor cells upon spheroid infiltration, associated with NKG2A increase on infiltrating immune cells. NKG2A-HLA-E pathway has already been described as a potential inhibitor of antitumor immune responses [45, 49]. In our settings, we observe that NKG2A blockade alone has no impact up to 48 h after coculturing immune cells and tumor spheroids, but that it could serve as a useful combinatorial treatment to avoid NKG2A-mediated tumor resistance to immunotherapy.

Our results are in line with other studies that used tumor spheroids to examine tumor-lymphocytes interactions in various contexts [26–32]. The specificity of our model resides in its setup simplicity and high versatility, that allows for multiple culture conditions and readouts to analyze dynamic responses in a controlled environment. To our knowledge, we are also one of the first group reporting the precise flow cytometry phenotyping of T and NK cells infiltrating human tumor spheroids, and the study of new immune modulators on human autologous antitumor responses.

We chose to focus on the response of T cells and NK cells in this context because these cells are the main effectors of antitumor immune response. Nevertheless, we observed that blood monocytes and dendritic cells are also able to efficiently infiltrate HT29 spheroids (data not shown). Heterotypic cocultures of tumor spheroids with other immune cells types could thus permit to expand our knowledge on human antitumor immune responses.

We adapted our allogeneic model to autologous cocultures derived from primary CRC tumor samples to generate a clinically relevant functional assay. Despite interindividual size variability, we were able to grow spheroids on a per-patient basis within 2 weeks. These spheroids were composed of mixed tumor-associated fibroblast and tumor cells (Additional file 1: Figures S5 and S6), thus mimicking tumor structure. Autologous cocultures with patients-derived spheroids showed that TILs were unable to reproducibly destroy autologous CRC tumors. This was expected, as tumors are edited to develop in front of an activated immune system that becomes unable to eradicate tumor cells [5]. However, we also showed that stimulating these TILs induce an immune response capable of destroying tumor structures. This has been observed using non-specific stimulation with IL-15 and more importantly using specific immune modulatory anti-MICA/B and anti-NKG2A monoclonal antibodies. Hence, spheroid cocultures are functionally relevant to the study of immunotherapies, echoing a recent study from Voest group [50], and pave the way for the study of anti-MICA/B and anti-NKG2A for cancer treatment. Our results also highlight the powerful antitumor potential of IL-15-based treatments for which cocultured spheroids could help deeply characterize the efficacy and mode of action.

## Conclusions

Overall, we show that human tumor spheroids cocultured with immune cells are relevant in vitro and ex vivo tools for human functional assays. As such, they will be a major addition to our scientific arsenal to study tumor immunology and will also help the scientific community to refine, reduce and complement animal experimentation. This field of research is rather new and thus widely opened to technical and scientific advances. Active research regarding the automation, miniaturization and adaptation of spheroid coculture models to all human tumor types will allow to dynamically study the antitumor immune response arising in patients and ultimately anticipate treatments efficacy in a personalized manner.

## Additional files

Additional file 1: **Figure S1.** Tregs infiltration in HT29 spheroids. Percentages of Foxp3+CD25+ Tregs among CD4+ T cells analyzed in Fig. 1c and d. **Figure S2.** IFNg blockade decreases spheroid infiltration and destruction by immune cells. (A) Pictures and analyses of (B) spheroid volume, (C) tumor cell apoptosis, and (D) spheroid infiltration 48h after coculturing HT29 spheroids with CD19-CD14- PBMCs in the presence or not of anti-IFNg blocking antibodies. **Figure S3.** T cell subsets and NKG2D expression by CD8 T cells after MICA/B treatment. (A) CD4 and (B) CD8 T cells proportions as well as (C) NKG2D expression by CD8 T cells relative to experiments in Fig. 5f to k. **Figure S4.** T and NK cells proportions and CD137 expression by CD8 T cells after combination therapy. Proportions of (A) T and NK cells, of (B) CD4 and CD8 T cells subsets and (C) CD137 expression by CD8 T cells relative to Fig. 6g to h. **Figure S5.** Pictures of primary CRC tumors cultures. Pictures of (A) primary CRC tumor cultured in adherent culture flasks and (B) tumor-derived spheroids used in autologous cocultures. **Figure S6.** Primary CRC-derived spheroids contains significant amount of EpCAM+ tumor cells. (A) Picture of primary CRC-derived spheroids and (B) flow cytometry or (C) IF analyses of EpCAM+ staining in the spheroids. **Table S1.** Clinical characteristics of the patients used for autologous cocultures. **Table 2.** Tumor cells content of the spheroids and T and NK cells composition of the TILs used for autologous cocultures. Percentages of tumor cells (EpCAM+CD45-) in patients-derived spheroids and percentages of NK cells (CD3e-CD56+) and T cells (overall CD3+, CD4 T cells CD3+CD4+CD8-, CD8 T cells CD3+CD4-CD8+) in respective autologous TILs used for cocultures. (DOCX 24846 kb)
