# Supplementary material for: Cocultures of human colorectal tumor spheroids with immune cells reveal the therapeutic potential of MICA/B and NKG2A targeting for cancer treatment
Source: J Immunother Cancer. 2019 Mar 14;7:74. doi: 10.1186/s40425-019-0553-9 (PMC6417026; doi:10.1186/s40425-019-0553-9)
Supplement: Supplementary file 4 — Figure S1. Tregs infiltration in HT29 spheroids. Percentages of Foxp3+CD25+ Tregs among CD4+ T cells analyzed in Fig. 1c and d. Figure S2. IFNg blockade decreases spheroid infiltration and destruction by immune cells. (A) Pictures and analyses of (B) spheroid volume, (C) tumor cell apoptosis, and (D) spheroid infiltration 48h after coculturing HT29 spheroids with CD19-CD14- PBMCs in the presence or not of anti-IFNg blocking antibodies. Figure S3. T cell subsets and NKG2D expression by CD8 T cells after MICA/B treatment. (A) CD4 and (B) CD8 T cells proportions as well as (C) NKG2D expression by CD8 T cells relative to experiments in Fig. 5f to k. Figure S4. T and NK cells proportions and CD137 expression by CD8 T cells after combination therapy. Proportions of (A) T and NK cells, of (B) CD4 and CD8 T cells subsets and (C) CD137 expression by CD8 T cells relative to Fig. 6g to h. Figure S5. Pictures of primary CRC tumors cultures. Pictures of (A) primary CRC tumor cultured in adherent culture flasks and (B) tumor-derived spheroids used in autologous cocultures. Figure S6. Primary CRC-derived spheroids contains significant amount of EpCAM+ tumor cells. (A) Picture of primary CRC-derived spheroids and (B) flow cytometry or (C) IF analyses of EpCAM+ staining in the spheroids. Table S1. Clinical characteristics of the patients used for autologous cocultures. Table 2. Tumor cells content of the spheroids and T and NK cells composition of the TILs used for autologous cocultures. Percentages of tumor cells (EpCAM+CD45-) in patients-derived spheroids and percentages of NK cells (CD3e-CD56+) and T cells (overall CD3+, CD4 T cells CD3+CD4+CD8-, CD8 T cells CD3+CD4-CD8+) in respective autologous TILs used for cocultures. (DOCX 24846 kb) [file 40425_2019_553_MOESM1_ESM.docx]

**Additional files**

**Figure S1: Tregs infiltration in HT29 spheroids.**

HT29 spheroids were cocultured with CD19-CD14- sorted PBMCs in the presence or not of IL-15. 24h later, we observed the percentages of Foxp3+CD25+ Tregs among CD4+ T cells IN and OUT the spheroids.

**Figure S2: IFNg blockade decreases spheroid infiltration and destruction by immune cells.**

(A) Representative pictures and analyses of (B) spheroid volume, (C) tumor cell apoptosis, and (D) spheroid infiltration 48h after coculturing HT29 spheroids with CD19-CD14- PBMCs in the presence or not of anti-IFNg blocking antibodies. n=5 independent experiments. Statistical significance was analyzed using paired t test (* p<0.05; ** p<0.005, *** p<0.001, **** p<0.0001).

**Figure S3: T cell subsets and NKG2D expression by CD8 T cells after MICA/B treatment.**

HT29 spheroids were cocultured or not with CD19-CD14- PBMCs in the presence of anti-MICA/B antibodies or corresponding control isotype. We analyzed (A) CD4 and (B) CD8 T cells proportions as well as (C) NKG2D expression by CD8 T cells by flow cytometry at 24h. n=10 independent experiments. Statistical significance was analyzed using the Wilcoxon matched-pairs signed rank test (* p<0.05; ** p<0.005, *** p<0.001, **** p<0.0001).

**Figure S4: T and NK cells proportions and CD137 expression by CD8 T cells after combination therapy.**

HT29 spheroids were cocultured or not with CD19-CD14- PBMCs in the presence of either anti-MICA/B antibodies alone or combined with anti-NKG2A blocking antibodies, or with corresponding control isotype antibodies alone or combined. We analyzed the proportions of (A) T and NK cells, of (B) CD4 and CD8 T cells subsets and (C) CD137 expression by CD8 T cells in the IN and OUT compartments by flow cytometry at 24h. Statistical significance was analyzed using the Wilcoxon matched-pairs signed rank test (* p<0.05; ** p<0.005, *** p<0.001, **** p<0.0001).


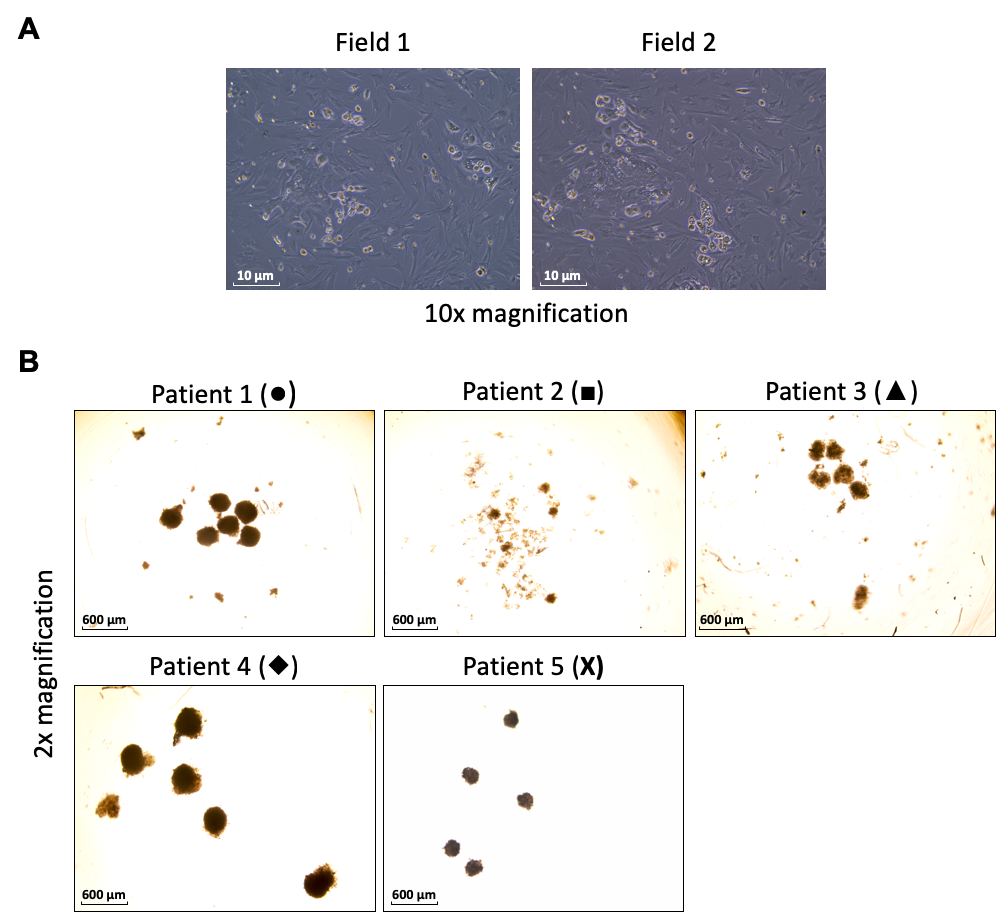


**Figure S5: Pictures of primary CRC tumors cultures.**

(A) Phase contrast pictures of primary CRC tumor cultured in classic adherent culture flasks. (B) Brightfield pictures of primary CRC tumor-derived spheroids used in the autologous cocultures.

**Figure S6: Primary CRC-derived spheroids contains significant amount of EpCAM+ tumor cells.**

(A) Brightfield picture of primary CRC-derived spheroids and (B) flow cytometry or (C) IF analyses of EpCAM+ staining in the spheroid.

**Table S1: Clinical characteristics of the patients used for autologous cocultures.**

This table presents clinical characteristics of the 5 patients for which we derived spheroids and performed autologous cocultures. These characteristics include age, gender, tumor localization, tumor cells differentiation, TNM grade, MSI/MSS status and mutational profile of BRAF, PI3K, KRAS and NRAS genes. Each patient is represented with distinct symbol.

**Table S2: Tumor cells content of the spheroids and T and NK cells composition of the TILs used for autologous cocultures.**

This table presents flow cytometry-measured percentages of tumor cells (EpCAM+CD45-) in patients-derived spheroids and percentages of NK cells (CD3e-CD56+) and T cells (overall CD3+, CD4 T cells CD3+CD4+CD8-, CD8 T cells CD3+CD4-CD8+) in respective autologous TILs used for cocultures.
